# Alternative immune checkpoints in immunoregulatory profile of cancer stem cells

**DOI:** 10.1016/j.heliyon.2023.e23171

**Published:** 2023-12-02

**Authors:** Keywan Mortezaee, Jamal Majidpoor

**Affiliations:** a Department of Anatomy, School of Medicine, Kurdistan University of Medical Sciences, Sanandaj, Iran; b Department of Anatomy, School of Medicine, Infectious Diseases Research Center, Gonabad University of Medical Sciences, Gonabad, Iran

**Keywords:** Immune checkpoint inhibitor (ICI), Cancer stem cell (CSC), Tumor microenvironment (TME), Alternative immune checkpoint (AIC), Resistance

## Abstract

Tumor-mediated bypass of immune checkpoint inhibitor (ICI) therapy with anti-programmed death-1 (PD-1), anti-programmed death-ligand 1 (PD-L1, also called B7–H1 or CD274) or anti-cytotoxic T lymphocyte associated antigen-4 (CTLA-4) is a challenge of current years in the area of cancer immunotherapy. Alternative immune checkpoints (AICs) are molecules beyond the common PD-1, PD-L1 or CTLA-4, and are upregulated in patients who show low/no ICI responses. These are members of B7 family including B7–H2 (ICOS-L), B7–H3 (CD276), B7–H4 (B7x), V-domain immunoglobulin suppressor of T cell activation (VISTA), B7–H6, HHLA2 (B7–H5/B7–H7) and catabolic enzymes like indoleamine 2,3-dioxygenase 1 (IDO1), and others that are also contributed to the regulation of tumor immune microenvironment (TIME). There is also strong evidence supporting the implication of AICs in regulation of cancer stemness and expanding the population of cancer stem cells (CSCs). CSCs display immunoregulatory capacity and represent multiple immune checkpoints either on their surface or inside. Besides, they are active promoters of resistance to the common ICIs. The aim of this review is to investigate interrelations between AICs with stemness and differentiation profile of cancer. The key message of this paper is that targeted checkpoints can be selected based on their impact on CSCs along with their effect on immune cells. Studies published so far mainly focused on immune cells as a target for anti-checkpoints. *Ex vivo* engineering of extracellular vesicles (EVs) equipped with CSC-targeted anti-checkpoint antibodies is without a doubt a key therapeutic target that can be under consideration in future research.

## Highlights

• AICs are upregulated following anti-PD-(L)1 or anti-CTLA-4.
• CSCs highly express AICs.
• Expression of AICs on CSCs control TIME.
• Targeting AIC-expressing CSC boosts immunotherapy outcomes.

## 1 Introduction

Developing resistance to immune checkpoint inhibitor (ICI) therapy is a critical issue in the area of cancer immunotherapy [1] in which there are increasing number of patients who are not responsive to such therapy [2]. This is indicative of the need for surveying factors contributed to the tumor-mediated bypass of ICI therapy. Alternative immune checkpoints (AICs) are considered as one possible contributor to such bypassing effects. In fact, tumors are equipped with complex systems containing a number of factors, rather than the common programmed death-1 (PD-1), programmed death-ligand 1 (PD-L1) and cytotoxic T lymphocyte associated antigen-4 (CTLA-4), that are also acting as immune checkpoints for hampering common ICI responses. Thus, upregulation of AICs can be one dominant reason in tumors bypassing ICI therapy. AICs are referred majorly to the other members of B7 family along with agents in the category of immune checkpoints but not belong to the B7 family.

There is strong evidence of the positive relation between cancer stemness with cancer-mediated bypass of ICI therapy [3,4]. Cancer stem cells (CSCs) are cells with self-renewal capacity that are harboring special tumor microenvironment (TME) niches and display high tumorigenic potential [5]. CSCs are considered as one of the key drivers of immunosuppression within TME. CSCs affect a number of cells within tumor immune microenvironment (TIME), and their disruption will cause tumor sensitization to therapy [6,7]. In this paper, we aimed to discuss about a possible link between activation of AICs with the stemness profile of cancer. Uncovering more about interactions among various immune checkpoints in the TIME and targeting key drivers of resistance is of particular importance and a hot topic of current investigations. To the best of our knowledge, this is the first review comprehensively investigating the impact of AICs in cancer stemness profile, selection of which can offer promising anti-checkpoint strategies to go with common ICIs for avoiding resistance and rendering durability of immunotherapy.

## 2 Cancer stem cells in cancer immunity and immunotherapy

CSCs are cells with self-renewal capacity that show increased fraction upon tumor progression. CSCs are designated through expressions of aldehyde dehydrogenase (ALDH), CD44 [8], CD133 [9], and BMI1. LGR5, Oct4, Sox9 and LSD1 are other known markers of cancer stemness [1]. CSCs interact with a number of cells within TIME [10]. CSCs show bidirectional cross-talk with tumor-associated macrophages (TAMs). CSCs stimulate pro-tumor type 2 macrophage (M2) recruitment [6] and promote macrophage differentiation toward M2 phenotype [7], and the activity of M2 macrophages further expands the population of CSCs [11]. CSCs also stimulate the activity of myeloid-derived suppressive cells (MDSCs) [12], and promote generation [13] and infiltration [14] of regulatory T cells (Tregs). In addition, CD44^+^CD133^+^ CSC expansion correlates inversely with CD8^+^ T cell expansion [3]. CSCs express the common PD-L1 checkpoint [15], and a positive link is identified between PD-L1 with expression of CSC markers [16]. Besides, CD44^+^ CSCs show resistance to adaptive T cell therapy through engaging CTLA-4/CD80 interaction [17]. Below, implication of immune checkpoints beyond the common PD-(L)1 and CTLA-4 in cancer stemness, immunoregulation and ICI responses are discussed.

## 3 The impact of alternative immune checkpoints on cancer stemness

**Fig. 1:**
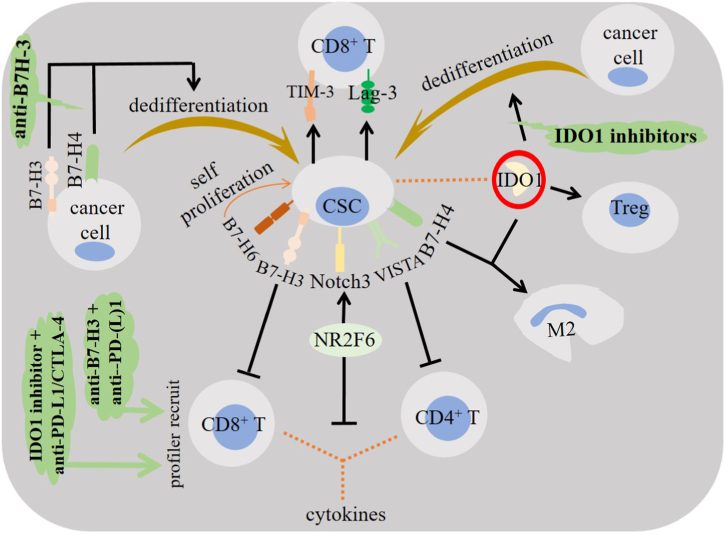
The impact of alternative immune checkpoints (AICs) on cancer stem cells (CSCs) and their immunoregulatory roles. Orphan nuclear receptor subfamily 2 group F member 6 (NR2F6), lymphocyte activation gene-3 (Lag-3), T cell immunoglobulin mucin-3 (TIM-3) and indoleamine 2,3-dioxygenase (IDO) are AICs that are upregulated in CSCs and their overexpression promote immune evasion. CSCs also overexpress a number of B7 family members in order to evade from therapy. Conversion of cancer cells into CSCs and increasing the expansion of such cells within tumor microenvironment (TME) are key functions directed by the impact of AICs. B7–H3 blockade eliminates CSCs through increasing the infiltration of CD8^+^ T cells, and IDO1 inhibition reduces CSC proportion. Thus, AICs can be targeted and used as a supplementary to the common immune checkpoint inhibitors (ICIs) in strengthening anti-tumor immunity. VISTA, V-domain immunoglobulin suppressor of T cell activation. M2, macrophage type 2; and Treg, regulatory T cell.

Expression of AICs in relation with cancer stemness is discussed and summarized in Table 1 and Fig. 1.

**Table 1:** Alternative immune checkpoint (AIC) expression profile in cancer stem cells (CSCs).

cancer type	CSC marker	AICs and their effect/s	Ref.
EOC model	Oct4, Sox2 & Nanog	NR2F6 overexpression associated with CSC phenotype through Notch3 signaling.	[18]
SCLC	CD44^+^ CD90^+^	CSCs upregulated Lag-3 and TIM-3 along with PD-(L)1 and CTLA-4.	[15]
cervical cancer	Oct4^+^ & Sox2^+^	IDO1 expression positively correlated with Notch1 signaling in CSCs.	[19]
human GBM	CD133^+^	B7–H2 upregulated along with PD-L1 and B7–H3 in CSCs from recurrent cancer.	[20]
prostate cancer mice	ALDH^+^CD44^+^	B7–H3 expressed at higher levels on CSCs (vs. bulk cancer cells) in cell lines under FIR. CSCs are seemingly more sensitive to B7–H3-targeted therapy.	[8]
CRC pts	CD133^+^	B7–H3 co-expression with CD133 associated with cancer metastasis and weaker patient survival.	[9]
HNSCC human & mice	BMI1^+^	B7–H3 highly expressed on CSCs and enabled the cells to escape immune surveillance.	[21]
breast cancer	CD44^+^	B7–H3 upregulation associated with CSC enrichment through its stimulatory effect on MVP/MEK activation.	[22]
gastric cancer pts	CD44^+^CD133^+^	B7–H3 associated with cancer stemness.	[23]
cervical cancer model	Oct4 & Sox2	B7–H3 expressed at high levels in sphere-forming tumor cells.	[24]
human ESCC	Oct4, Sox9, LGR5 & LSD1	B7–H4 associated positively with expression of cancer stemness markers and higher tumor immaturity profile.	[25]
CRC pts	CD44^+^CD133^+^	B7–H4 co-expressed with CD44^+^CD133^+^ cells in tumor tissues and associated with EMT profile of cancer.	[26]
pancreatic cancer pts	CD44^+^	B7–H4 inhibition reduced CD44 expression in tumor cells.	[27]
glioma xenograft	CD133^+^	B7–H4 expressed by CD133^+^ and CD133^-^ cells.	[28]
pancreatic and breast cancers	–	VISTA expressed at lower level in well-differentiated but higher in poorly differentiated cancer, with the latter being the type of cancer where CSCs are commonly found.	[10,29,30]
glioma pts	Sox2^+^ CD133^+^	B7–H6 co-expressed with Sox2^+^ cells in tumor tissues, and its abnormal level associated with CSC proliferation.	[31]

EOC, epithelial ovarian cancer; SCLC, small cell lung cancer; pts, patients; ALDH, aldehyde dehydrogenase; FIR, fractionated irradiation; HNSCC, head and neck squamous cell carcinoma; CRC, colorectal cancer; EMT, epithelial-mesenchymal transition; MVP, major vault protein; GBM, glioblastoma; ESCC, esophageal squamous cell carcinoma; Lag-3, lymphocyte activation gene-3; TIM-3, T cell immunoglobulin mucin-3; VISTA, V-domain immunoglobulin suppressor of T cell activation; and IDO1, indoleamine 2,3-dioxygenase 1.

### 3.1 Nuclear receptor subfamily 2 group F member 6

Orphan nuclear receptor subfamily 2 group F member 6 (NR2F6) is an intracellular immune checkpoint that acts for hampering transcription thresholds of cytokines by CD4^+^ and CD8^+^ effector T cells [32]. There is a report of a positive link between NR2F6 overexpression with promotion of CSC phenotype in epithelial ovarian cancer, mediated through activation of Notch3, a signaling involved in DNA damage repair and mediating CSC anti-apoptotic activity [18].

### 3.2 Lymphocyte activation gene-3 and T cell immunoglobulin mucin-3

Lymphocyte activation gene-3 (Lag-3, or CD223) exerts regulatory roles on T cell activity to promote immune escape [33]. T cell immunoglobulin mucin-3 (TIM-3) is another checkpoint that promotes exhaustive state in T cells, and is considered as a compensatory mechanism for permitting tumor escape from immune system [[34], [35], [36]]. Galectin-3 and galectin-9 are the respective ligands for Lag-3 and TIM-3 that are expressed on CSCs. CD44^+^ CD90^+^ CSCs from small cell lung cancer (SCLC) show upregulation of Lag-3 and TIM-3 along with overexpression of the common checkpoints PD-(L)1 and CTLA-4. CSCs express such checkpoints upon interaction with CD8^+^ T cells. Lag-3, TIM-3 and PD-1 positive T cells are also identified in proximity to CSCs [15], which is indicative of the checkpoint-mediated regulatory roles in CSC suppressive effect on Teff activity [37].

### 3.3 Indoleamine 2,3-dioxygenase

Indoleamine 2,3-dioxygenase (IDO) is among endogenous immune checkpoints and an active promoter of immunosuppression. IDO promotes MDSC recruitment and activation [38], inhibits T cell responses [39,40], and promotes Treg-M2 co-operative activities [41]. There is compelling evidence of a possible implication of IDO1 and its downstream metabolites in self-renewal maintenance of embryonic stem cells [42]. A positive correlation between Notch1 signaling with IDO1 expression is also identified in cervical CSCs [19]. As the product of IDO1, kynurenine increases expression of the CSC markers Oct4 and Sox2 and enhances formation of tumorosphere capacity [19]. Kynurenine signaling molecule inhibitors dampened CSC potential of cancer cells [43]. Glioblastoma (GBM) CSC-derived extracellular vesicles (EVs) containing low amount of PD-L1 represent high levels of IDO1, which is suggestive of multiple layers of immune regulation in such tumor type [44]. Enhanced WNT/β-catenin activity within TME defines stemness feature in cancer cells [45,46] and is associated with increased IDO1 representation in dendritic cells (DCs) [47]. This implies that targeting endogenous immune checkpoints like IDO1 can be an effective way for hampering cross-talk between CSCs with immune cells within TIME, which may lead to durable and more effective outcomes.

### 3.4 B7-homolog 2

B7-Homolog 2 (B7–H2, also called ICOS-L) is a ligand for inducible costimulatory (ICOS) molecule expressed on T cells [48]. B7–H2 expression in hepatocellular carcinoma (HCC) patients correlates with poor clinical outcomes [49]. Upregulation of B7–H2 along with PD-L1 is reported in CSCs derived from recurrent GBM compared with cells from primary tumor [20]. This suggests that B7–H2 and PD-L1 may co-contribute to the immunosuppressive activity of CSCs and to a cold immunity of a cancer like GBM.

### 3.5 B7-homolog 3

B7-Homolog 3 (B7–H3, also called CD276 or B7RP-2) is a type-1 transmembrane glycoprotein and an immunoregulatory molecule that shows limited expression in most normal tissues [9,50,22]. By contrast, B7–H3 is overexpressed in a number of human cancers including oral cancer [51], colorectal cancer (CRC) [52], HCC [53] and prostate cancer [54], and it acts as a potent suppressor of T cell activation [55]. B7–H3 is highly expressed on CSCs [50], and its expression correlates with cancer stemness [23]. High B7–H3 expression is reported in 76 % of specimens collected from GBM patients [56], and its upregulation occurs particularly in CSCs derived from recurrent GBM [20]. In non-small cell lung cancer (NSCLC), a positive correlation between tumoral B7–H3 with poor differentiation and nodal metastasis is identified [57]. B7–H3 expression is also reported in 22/28 patients with CD133^+^ CRC, and its co-expression with CD133 is associated with tumor metastasis and weaker patient survival [9]. Prostate cancer cell lines under fractionated irradiation showed upregulation of B7–H3 checkpoint on bulk cells and CSCs, which was lasted for up to 3 days. The expression of this checkpoint is naturally higher on prostatic CSCs compared with bulk cancer cells [8]. It seems that the expression of B7–H3 on cancer cells is for promoting an epithelial-mesenchymal transition (EMT) phenotype, thereby acquiring a stemness profile in such cells [58]. A variety of phenotypical states are found in bladder cancer. A report in this area showed no expression of CD24, but B7–H3 and CD44 were identified at metastatic lymph node area [59]. Interpretations from a study in this area imply the population of CD24^low^CD44^high^ cells as CSCs, while CD24^high^CD44^low^ cells are regarded as non-CSC population [22]. B7–H3 is used by CSCs to evade from immune surveillance, and is considered as a functional marker for isolation of CSCs from squamous cell carcinoma (SCC) [21]. In bladder cancer, the population of CD276 ^high^ CSCs are slow proliferating and may evade from immune surveillance [59].

### 3.6 B7-homolog 4

B7-Homolog 4 (B7–H4, also called B7S1, VTCN1, and B7x) is another checkpoint that its overexpression is indicative of poor prognosis [60] and is linked with cancer progression [61,62]. B7–H4 is expressed generally on T cells and antigen-presenting cells (APCs) [25]. B7–H4 is involved in the expansion of Foxp3^+^ Tregs [63] and is a negative regulator of T cell immunity [64] in which proliferation and activity of both CD4^+^ and CD8^+^ T cells are suppressed by B7–H4 [27]. A positive association is identified between B7–H4 with an EMT profile in CRC cells, and with CSC characteristics, more aggressive behavior of tumor and poor patient survival [26]. EMT is a *trans*-differentiation state that its presence in a tumor is indicative of CSC expansion and more aggressive behavior [65]. B7–H4 expression along with Sox9, Oct4, and LSD1 in esophageal SCC is suggestive of considering this checkpoint as a novel marker of CSCs and its importance in prognostic evaluation of cancer patients [25]. B7–H4 has a role in directing cross-talk between CSCs with TAMs, which is indicative of weak prognosis in glioma patients [66]. From mechanistic overview, a positive link is identified between hypoxia and hypoxia inducible factor (HIF)-1α with cytoplasmic B7–H4 expression in tumor cells [67]. This infers that TME conditions like hypoxia can affect B7–H4 regulation and tumor stemness.

### 3.7 V-domain immunoglobulin suppressor of T cell activation

V-domain immunoglobulin suppressor of T cell activation (VISTA, also called programmed death-1 homolog [PD-1H]) is an immune regulatory and inhibitory checkpoint that checks immune and tumor cells in cancer immunotherapy [68]. Upregulation of VISTA occurs secondary to the anti-PD-(L)1 or anti-CTLA-4 [69], and its expression on tumor cells is contributed to the regulation of T cell functionality [70]. This is mediated through promoting naïve T cell quiescence [71] and hampering CD4^+^ T cell responses [72]. Outcomes of a study on human breast cancer showed higher rate of VISTA expression on poorly differentiated cancer type [29], and in pancreatic cancer it was found lower levels of VISTA in tumors with more differentiated neoplasm [30]. Due to that CSCs are commonly found in poorly differentiated tumors [10], it is fair to announce that in patients with high proportion of CSCs, high VISTA expression is presumably considered as a mechanism of immune evasion and immunotherapy resistance. Thus, strategies to hamper increased VISTA activity in poorly differentiated cancers can offer promising combinatory to anti-PD-(L)1 in cancer immunotherapy.

### 3.8 B7-homolog 6

B7-Homolog 6 (B7–H6) promotes human natural killer (NK) cell functionality through activation of NKp30 [73], which is a natural marker of NK cell functionality [74]. B7–H6 displays abnormal expression in cancer. In glioma, B7–H6 is expressed preferentially in CSCs, delineated by co-expression of B7–H6 with Sox2 in tumor tissue [31], so it can be a target for increasing immunogenicity of such cancer type.

### 3.9 B7-homolog 7

B7-Homolog 7 (B7–H7, also called HHLA2) is the last member of B7 family that acts via attachment to CD28H (IGPR1 or TMIGD2) receptor [75]. HHLA2 is upregulated in HCC [76], and positively correlated with more advanced stage, poor differentiation state, microvascular invasion and worse prognosis of such cancer type [77]. HHLA2 knockdown in clear cell renal cell carcinoma (ccRCC) is reported to significantly associated with reduced vimentin and N-cadherin expressions and increased E-cadherin expression, which is indicative of a positive correlation between HHLA2 with cancer stemness profile of cancer [78]. By contrast, in epithelial ovarian cancer, HHLA2 expression is found to significantly correlated with cancer cell differentiation in which higher HHLA2 level was identified in well-differentiated cancers. It was also attested a positive association between HHLA2 with increased density of CD8^+^ T cells in this cancer type [79]. In pancreatic and ampullary cancers, 50 % of patients with low expression of HHLA2 had poorly differentiated cancer, while about 18 % of highly expressed HHLA2 cases had this cancer phenotype. Patients with high HHLA2 expression had better post-surgical survival and delayed tumor recurrence [80]. Variation in the activity of HHLA2 is indicative of the potential of this checkpoint to act as both inhibitor and activator of immune system through involving different types of receptors. CD28H is the co-stimulatory receptor in HHLA2 signaling that can be a target for agonistic antibodies for inducing the effector side of anti-tumor immunity [81].

## 4 From therapeutic standpoint

Contribution of AICs to powering stemness profile of cancer is indicative of their targeting as a promising supplementary strategy in cancer immunotherapy. Intensive correlation of CSCs with cancer metastasis, resistance and recurrence, and targeting AICs implicated in the enrichment of CSCs can define complementary therapeutic protocols with better and more durable outcomes, particularly in tumors with cold immunity. This area is under recent focus in cancer immunotherapy. Unfortunately, studies published so far on involving CSCs for evaluating therapeutic efficacy of anti-AIC therapy are uncommon. This can be a challenge to direct our interpretations toward clinical practice. However, limited number of studies offer hopes in this area so that our focus in cancer immunotherapy needs to be expanded and not just limited to targeting some immune cells in TME, but also considering CSCs as one of the key cancer promoting cells in such milieu. There is a study attesting IDO1 downregulation in cervical CSCs after Notch1 inhibition [19]. Application of the IDO1 inhibitor LW106 resulted in the substantial elevation of Teff infiltration and reduced recruitment of Tregs, and in xenograft tumor models resulted in the reduced proportion of CSCs [82]. These are indicative of targeting AICs as a way for recovering anti-tumor immunity and possibly expanding the durability of therapy. In regard with B7–H3, expression of this checkpoint on both differentiated cancer cells and CSCs with limited heterogeneity along with its expression on tumor vasculature and stroma and its restricted distribution among normal organs are all indicative of the importance of application of monoclonal antibodies (mAbs) against this glycoprotein for the sake of better control over TME and promoting a durable therapy [58]. Thus, targeting B7–H3 can be a promising strategy for removal of both CSCs and differentiated tumor cells [56]. There is a report of a more potent cytotoxic potential of B7–H3-targeting chimeric antigen receptor (CAR) T cell therapy on CSCs (vs. bulk cancer cells), indicating the higher sensitivity of B7–H3^+^ CSCs to B7–H3-targeting therapy [8]. B7–H3 inhibition eliminates CSCs through promoting the anti-tumor activity of CD8^+^ T cells [21]. 376.96 is a mAb that identifies B7–H3 on human ovarian cancer cells and CSCs, and its application in A2780 ovarian cancer is reported to reduce the population of ALDH^+^ CSCs by 50 % [83]. Targeting B7–H4 can also be considered as a suggested strategy in cancers with high CSC profile. In human pancreatic cancer, for instance, inhibition of B7–H4 is reported to reduce the expression of CD44, which is a marker of CSCs [27]. In the context of B7–H6, knockdown of this checkpoint using siRNAs in glioma CSCs resulted in their hampered proliferation profile through downregulation of c-Myc and repressing RNA guanine-7 methyltransferase (RNMT) activity [31]. Finally, application of agonistic CD28H antibodies can possibly reduce the proportion of CSCs and boost the power of immune system against cancer. It can also be considered for developing bispecific antibodies in ICI therapy of cancer [81] (Fig. 1).

## 5 Conclusions and future perspectives

**Fig. 2:**
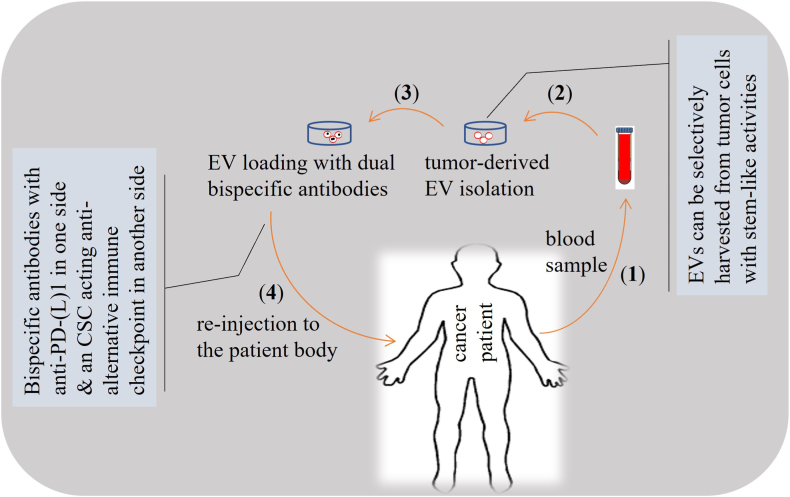
Implication of extracellular vesicles (EVs) for delivery of bispecific antibodies into tumor area. Here, tumor-derived EVs are extracted from blood and loaded with bispecific antibodies that have one inhibitor of a common checkpoint including anti-programmed death-1 (PD-1)/anti-programmed death-ligand 1 (PD-L1) in one side and an inhibitor of alternative immune checkpoint (AIC) in another side, particularly selecting AICs with activity on cancer stem cells (CSCs). The rationale for this targeted delivery is that tumor-derived EVs are attracted preferentially toward tumor area, and selection of CSC-targeted anti-checkpoints will reduce resistance rate and increase therapeutic durability.

AICs are promising targets in cancer immunotherapy, as they are linked positively with CSC expansion and activity (depicted in Table 1). Expanding the proportion of CSCs and boosting their cross-talk with cells within TIME is a key mechanistic description for the negative impact of AICs on cancer immunotherapy. Targeting AICs can be considered as a novel strategy and complementary to the common ICI therapy for reinvigorating anti-cancer side of tumor immunity, reducing tumor recurrence and exerting durable outcomes. A special focus is suggested in future studies on *ex vivo* engineering of EV-derived CSCs and their equipment with desired antibodies against the mentioned AICs. This strategy can offer promising and durable anti-tumor effects, and can even be considered as a promising supplementary to the common ICI therapy. As studies in this area are limited, so it is hard to assert for sure the significance of such treatment schedule, but there are evidences favoring the application of EVs as a novel approach for specific delivery of target drugs toward tumor area due to expressing tumor receptors on their surface, and also representing high durability upon systemic delivery because of the presence of covering membrane around their contents. Thus, the need for drug volume and, subsequently, the rate of adverse events will be reduced tremendously. Thus, EVs can be loaded with desired agents to increase the efficacy of immunotherapy [84]. A suggestion is to use dual checkpoint inhibitors using bispecific antibodies. This novel strategy can be used to simultaneously target one common checkpoint in one side, such as anti-PD-1/PD-L1 or anti-CTLA-4, and an AIC, particularly the one acting on CSCs, in another side. Such dual inhibitor bispecific antibodies can be packed in EVs for their targeted delivery (Fig. 2).

## Data availability statement

No data was used for the research described in the article.

## CRediT authorship contribution statement

**Keywan Mortezaee:** Writing – review & editing, Writing – original draft, Visualization, Supervision, Conceptualization. **Jamal Majidpoor:** Writing – review & editing, Writing – original draft.

## Declaration of competing interest

The manuscript entitled ‘Alternative immune checkpoints in immunoregulatory profile of cancer stem cells’ is the representative of novel insights toward the impact of alternative immune checkpoints in cancer stemness profile and using the strategy in improving cancer immunotherapy outcomes. All figures are originally stated and the idea is novel, and both authors are contributed significantly to the subject.
